# Knowledge and Opinions of French Dental Students Toward Occlusal and Proximal Restorative Thresholds

**DOI:** 10.3290/j.ohpd.b1749707

**Published:** 2021-07-15

**Authors:** Marie-Agnès Gasqui, Laurent Laforest, Justine Le Clerc, Romain Ceinos, Florence Chemla, Valérie Chevalier, Pierre Colon, Florence Fioretti, Alexis Gevrey, Olivia Kérourédan, Delphine Maret, Caroline Mocquot, Canan Özcan, Bruno Pelissier, Fabienne Perez, Elodie Terrer, Yann-Loïg Turpin, Reza Arbab-Chirani, Sophie Doméjean, Dominique Seux

**Affiliations:** a Lecturer, Multimaterials and Interfaces Laboratory, Faculty of Dentistry, University of Lyon 1, Lyon, France; Lyon University Hospital, Department of Conservative Dentistry, Lyon, France.; b Lecturer, Faculty of Dentistry, University of Lyon 1, Lyon, France.; c Lecturer, Multimaterials and Interfaces Laboratory, Faculty of Dentistry, University of Rennes, Rennes, France.; d Lecturer, Faculty of Dentistry, Côte d’Azur University, Nice, France; Hospital St Roch, Odontology Center, Nice, France; Bio-cultural Anthropology, Ethics and Health Law, Aix-Marseille University, France.; e Professor, Faculty of Dentistry, University Paris Descartes; Department of Dentistry, Charles Foix Hospital, APHP Paris, France.; f Lecturer, University of Bretagne Occidentale, Brest, France; Dental University Hospital of Brest, France.; g Professor, Paris University, AP-HP Rothschild Hospital Paris; University of Lyon, Lyon 1; Multimaterials and Interfaces Laboratory, Villeurbanne, France.; h Lecturer, Faculty of Dentistry, Strasbourg University, Department of Medicine and Dental Surgery, Strasbourg University Hospital,Strasbourg, France.; i Lecturer, Faculty of Dentistry, UFR Nancy, France.; j Lecturer, Faculty of Dentistry, University of Bordeaux, Bordeaux, France; University Hospital of Bordeaux, Department of Dental Surgery, Bordeaux, France; INSERM, Tissue Bioengineering, Bordeaux, France.; k Lecturer, Faculty of Dentistry Toulouse, University of Toulouse, AMIS Laboratory, Toulouse, France.; l Lecturer, Paris University, AP-HP Rothschild Hospital Paris; University of Lyon, Lyon 1; Multimaterials and Interfaces Laboratory, Villeurbanne, France.; m Lecturer, Department of Conservative Dentistry, Faculty of Dentistry, University of Reims Champagne-Ardennes, France.; n Doctor, Faculty of Dentistry, University of Montpellier, France.; o Professor, Inserm, Regenerative Medicine and Skeleton, ONIRIS, University of Nantes, Faculty of Dentistry, University Hospital, Nantes, France.; p Professor, Faculty of Dentistry, University Hospital Institute, Mediterranean Region Infectious Diseases, Marseille, France.; q Lecturer, University of Rennes, University Hospital of Rennes, Department of Conservative Dentistry, Rennes, France.; r Professor, Faculty of Dentistry and Dental University Hospital, University of Brest, Brest, France.; s Professor, University of Clermont Auvergne, Faculty of Dentistry; Research Center for Clinical Dentistry, Clermont-Ferrand, France; University Hospital Estaing Clermont-Ferrand, Dental Services, Clermont-Ferrand, France.; t Professor, Multimaterials and Interfaces Laboratory, Faculty of Dentistry, University of Lyon 1, Lyon, France; Lyon University Hospital, Department of Conservative Dentistry, Lyon, France.

**Keywords:** carious lesions, dental education, dental students, minimal intervention, restorative threshold

## Abstract

**Purpose:**

To investigate the practices, knowledge and opinions of French dental students (FDSs) in various domains of minimal intervention (MI) in cariology.

**Materials and Methods:**

A cross-sectional, questionnaire-based study was conducted in spring 2018 among all fifth-year French dental students (FDSs) from the 16 French dental schools. The present article focuses on restorative management. Statistical analyses (descriptive, chi-squared) were performed.

**Results:**

The response rate was 84.5%. Overall, 97.4% of respondents would have operatively intervened for proximal and 83% for occlusal carious lesions, respectively, while non-or micro-invasive intervention would have been possible. Interestingly, 15% would completely open the occlusal fissures. For both occlusal and proximal lesions requiring a restoration, composite resin was indicated by over 95% of the respondents. In a clinical case, 51.6% of FDSs who rightly diagnosed an enamel carious lesion would operatively intervene. When FDSs could not diagnose the type of carious lesions, a high proportion of invasive actions were also reported (40%). FDSs who read scientific articles were more likely to consider the high importance of not filling sound teeth unnecessarily (p = 0.033).

**Conclusion:**

FDSs do not have sufficient awareness of MI guidelines regarding occlusal and proximal restorative thresholds. Efforts are required in dental schools to teach FDSs to postpone invasive/restorative strategies to later stages of carious progression. There is a need to strengthen prevention techniques and non-invasive options in the teaching of MI in cariology.

Contemporary approaches to caries management have dramatically changed during the last decades. The treatment of caries involves assessment and management of risk factors.^8^ Preventive and non-invasive options based on individual caries risk must be favoured; restorative strategies must be postponed to later stages of lesion progression and must respect tooth structure and pulp vitality as much as possible.^6,7,11,16,22,24,30,31^ Minimal intervention (MI) in cariology is nowadays part of the latest recommendations for dental curricula.^5,22,25,34^ According to a recent expert Delphi consensus,^32^ non-cavitated carious lesions should be managed non- or micro-invasively, as should early cavitated carious lesions when the area around the lesion is easily accessible for cleaning. In contrast, cavitated lesions which are not cleansable usually require invasive/restorative management to restore shape, function and aesthetics.

Several questionnaire surveys have investigated the French general practitioners’ (GPs) knowledge of and attitudes towards cariology.^9,10,15^ They all showed that, despite improvements in recent years,^9^ MI in cariology has not been fully integrated into everyday clinical dental practice. Moreover, the authors highlighted differences in the GPs’ knowledge, perception and practice of MI with respect to different factors, notably gender, or reading specific articles on MI. Further, professional practice evolution is inherently linked to undergraduate and post-graduate education.^23^

Although many studies have examined the content of teaching in cariology, particularly in operative/restorative dentistry,^4,5,14,18,20,27-29^ there is a lack of data related to the direct effect of undergraduate education in cariology on the knowledge, opinions and treatment attitudes of dental students (DSs). It is not clear whether MI concepts are currently acquired by FDSs.

This study was the first of its kind in France. Its purpose was to investigate the knowledge and opinions of French DSs (FDSs) at a national level on several areas of MI in cariology, namely, caries risk assessment, dental sealants (preventive and therapeutic), restorative thresholds, and strategies for proximal and occlusal lesions as well as deep-lesion management. The present publication focuses on restorative thresholds and management strategies for proximal and occlusal carious lesions.

## Materials and Methods

Study methods have already been described in a recent article based on the same set of data, but focusing on caries risk and dental sealants.^19^ Briefly, a questionnaire survey was administered during the spring of 2018 to fifth-year FDSs of all 16 French dental schools (Bordeaux, Brest, Clermont-Ferrand, Lille, Lyon, Marseille, Montpellier, Nancy, Nantes, Nice, Paris Descartes, Paris Diderot, Reims, Rennes, Strasbourg and Toulouse). This project was institutionally supported by the Collège National des Enseignants en Odontologie Conservatrice (CNEOC; French national association of teachers in conservative dentistry). Printing and postal-mailing costs were sponsored by Colgate France.

### Study Population and Questionnaire Administration

This cross-sectional study involved all fifth-year FDSs (penultimate year before graduation) of the 16 French dental schools (N = 1370). After explaining the purpose of the study, FDSs were invited to complete a questionnaire during a course specifically organised for this purpose. As the questionnaire only concerned the learning outcomes, no ethics committee approval was required, according to the French regulation.

A questionnaire was self-administered (paper format; 18 pages) to the FDSs. The content of the different sections of the questionnaire is described in detail in the original articles.^9,10,15,19,33,35^ It consisted of several question formats (yes/no questions, closed-ended questions with forced choice or multiple allowable answers, and open-ended questions with open-ended writing).

The present manuscript refers to questions related to restorative thresholds and management strategies for proximal and occlusal carious lesions, with two clinical cases of minor or questionable occlusal lesions (based on occlusal view and radiographs), and to beliefs about selected aspects of carious lesion diagnosis and treatment.^9,35^

### Data Capture and Analysis

Data were entered into Excel spread sheets by four people (three dentists [DS, MAG, SD]). Descriptive and statistical analyses were performed with SPSS (IBM SPSS Statistics Version 19; Armonk, NY, USA). The present analyses were mainly descriptive. Missing data were not taken into account in the present study. A chi-squared test was used to assess 1. the associations between responses related to restorative thresholds and management strategies for proximal and occlusal lesions, and 2. gender and additional reading of scientific articles about MI in cariology. The level of statistical significance was set at 5%.

The analysis comprised two main parts: questions related to restorative dentistry and questions related to two clinical cases of minor or questionable occlusal carious lesions.

#### Questions related to restorative dentistry

- Proximal restorative threshold: enamel lesions (grades 1 and 2) vs lesions at the enamel-dentin junction (EDJ) (grade 3) vs lesion involving the outer third of dentin (grade 4) vs deeper lesions (grades 5 and 6).
- Occlusal restorative threshold: enamel lesions (grades 1 and 2) vs deeper lesions (grades 3-5); FDSs were asked to indicate at which level they would intervene invasively.

#### Questions related to two clinical cases of minor or questionable occlusal carious lesions

Management options were subdivided into: non-invasive strategies (topical fluoride application and therapeutic sealants) vs invasive strategies (restoration placement after cavity preparation limited to removal of carious tissue only vs cavity preparation limited to removal of carious tissue combined with sealant vs cavity preparation involving the whole fissure).

Both clinical cases were supposed to be at low caries risk.

## Results

All 16 French dental schools participated in the survey. Among the 1370 five-year FDSs, a total of 1158 completed the questionnaires (mean age: 24.5; 53.5% women), leading to a response rate of 84.5% (from 32.9% to 100% according to dental schools). Non-participants were FDSs who were absent the day of the survey. Over one-third of the respondents (34.8%) had already read scientific publications about MI in cariology.

### Restorative Threshold, Cavity Preparation and Material

#### Proximal carious lesions

**Fig 1:**
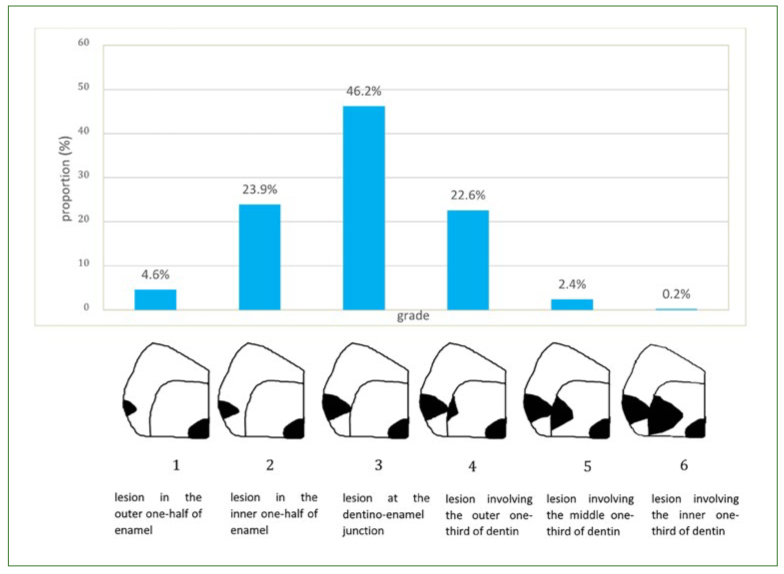
The earliest stage of proximal carious development, as assessed by radiography, at which the respondents (n = 1149) would intervene surgically.

A total of 74.7% of the respondents considered that restorative treatment (cavity preparation and restoration placement) is appropriate for a carious lesion confined to enamel reaching or not the EDJ (grades 1-3) (Fig 1), while 22.6% would wait for the lesion to reach the outer third of dentin (grade 4). FDSs who had read scientific articles would postpone their restorative threshold to the dentin level statistically significantly more often than would students who had not read scientific articles (p = 0.028). The preferred cavity designs for the smallest lesion requiring immediate restoration were tunnel (48.7%) or saucer-shaped (46.8%) preparations. However, 4.5% of respondents chose traditional Black class II preparations. FDSs who had read scientific articles were more interested in tunnel preparations, while those who did not were more likely to choose a saucer-shaped cavity design (p = 0.03). Responses varied according to gender; indeed, men were more likely to choose Black’s cavity preparation method than were women (p = 0.026). The preferred dental material was composite resin for almost all respondents.

#### Occlusal carious lesions

**Fig 2:**
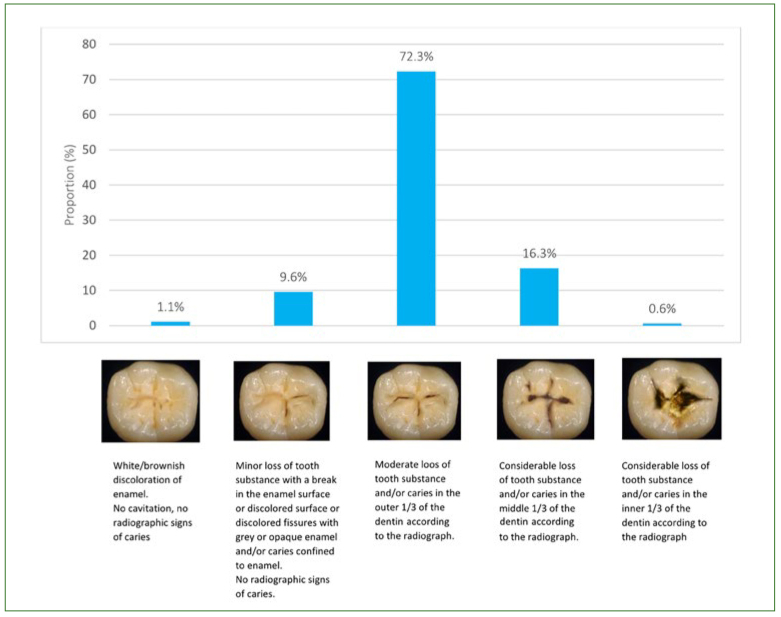
The earliest stage of occlusal carious development, as assessed clinically and radiographically, at which the respondents (n = 1156) would intervene with operative treatment.

Overall, 10.7% of the respondents would restore a lesion confined to enamel (grades 1 and 2). Nearly three out of four respondents (72.3%) would place their restorative threshold at the outer third of the dentin (grade 3). Lastly, 16.9% would wait for the lesion to reach the middle third of the dentin or even deeper (Fig 2). A large majority of respondents (83.5%) would limit the preparation to the lesion, while 15% would involve the entirety of occlusal fissures. Almost all respondents (98.9%) would opt for the use of a composite resin.

### Diagnosis and Treatment Alternatives for Minor or Questionable Occlusal Carious Lesions

#### Tooth 1

**Fig 3:**
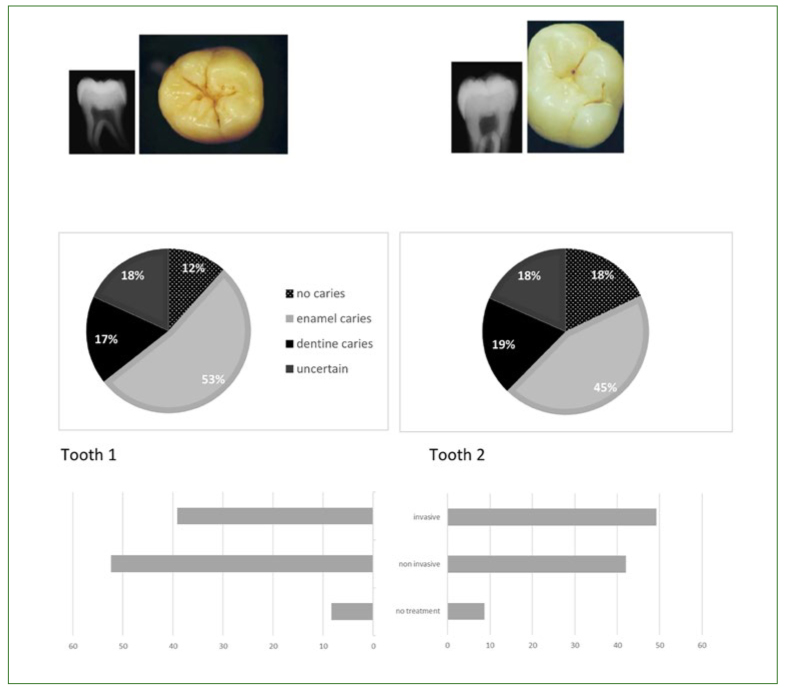
Respondents’ diagnoses (pie charts) and treatment suggestions (bar charts) for teeth 1 and 2 (number of respondents: n = 1132 for tooth 1; n = 1138 for tooth 2).

Respondents varied markedly in their diagnosis: half of the respondents (52.7%) diagnosed an enamel lesion and 17.4% a dentin lesion, while 18.2% did not identify any lesion and 11.7% were uncertain (Fig 3). About 39% opted for operative treatment, whereas 52.4% chose non-invasive strategies involving fluoride and remineralisation, and 8.4% would not propose any treatment.

Among the FDSs who diagnosed the presence of an enamel lesion, 62.4% would indicate non-invasive strategies, while 34% would perform a cavity preparation and place a restoration (3.6% would not treat). Among those who were uncertain about the presence/absence of a lesion, 32.1% would intervene surgically.

#### Tooth 2

The diagnostic variability observed for tooth 1 was also found for tooth 2; indeed, 44.5% of respondents believed that tooth 2 presented an enamel carious lesion, whereas 19.2% diagnosed the presence of a dentin lesion. 17.9% stated that the occlusal surface was sound and 18.4% remained uncertain.

Nearly half of the respondents (49.2%) would indicate operative treatment. The others (42.1%) would indicate non-invasive treatment (fluoride application: 11.7%; sealant: 30.4%) and a minority (8.7%) would not propose any treatment.

Therapeutic options differed according to gender: men would choose statistically significantly more therapeutic abstention and women more restoration placement (p = 0.001). For tooth 1, 51.6% of FDSs who diagnosed the presence of an enamel lesion would drill and fill. Likewise, among those who were uncertain about the presence/absence of a lesion, 40% would also choose an invasive option.

### Knowledge of FDSs on the Caries Process

The vast majority of the respondents (85.6%) believed that radiographs tended to underestimate the depth of a lesion compared to clinical findings. There was considerable variability in responses with regard to the time it takes for a proximal lesion to progress from the outer enamel to the EDJ. Indeed, 23.8% thought that a duration of between 7 and 11 months was necessary, whereas 24.6% reported over 12 months. A proportion of 30.1% of FDSs would monitor a radiographically detected lesion near the EDJ for six months to determine whether this lesion was active and to evaluate its rate of progression. In contrast, 51.8% would not, while 18.1% were uncertain. Men would monitor lesions statistically significantly less frequently than women (p = 0.022). While 40.7% of the respondents agreed that a proximal lesion at the EDJ has a visible cavitation, 37.1% disagreed and 22.2% were uncertain. When asked about the relative importance of under- and over-treatment, the majority of FDSs (58%) answered that these risks of error were of equal importance, although 28.1% thought that it was more important not to fill sound teeth unnecessarily (accepting the risk of not restoring some carious lesions), and 13.9% stated that it was more important to fill all carious teeth (accepting the risk of some unnecessary restorations). Answers statistically significantly differed according to the reading or not of scientific articles; specifically, those who read scientific articles were more likely to find it more important not to fill sound teeth unnecessarily than were those who did not (p = 0.033).

## Discussion

The present study, the first of its kind in France and worldwide, provides an overview of FDSs knowledge and behaviours regarding MI in cariology, more specifically in the context of proximal and occlusal carious lesions. All 16 French dental schools participated in the present study. Given the global satisfactory response rate (84.5%), it can be postulated that the results are highly representative of the knowledge and opinions of all fifth-year FDSs at the time of the study.

MI principles appeared to be partially integrated by FDSs for both proximal and occlusal carious lesion management.

When considering the proximal restorative threshold (Fig 1), 46.2% of the respondents placed it in grade 3 (at the EDJ), while a noticeable proportion of them tended to be even more invasive, as they operatively intervened for grade 1 (4.6%) and 2 (23.9%) lesions. It appears that these attitudes do not correspond to current guidelines recommending non- and micro-invasive interventions for lesions that have not progressed to the outer third of the dentin, in the absence of visible cavity.^32^ Although lesions confined to enamel are rarely cavitated (around 10%), the presence of cavitation increases when they reach EDJ and beyond (40% of cavitation for a radiolucency within external half of dentin).^26,32^ The presence of any cavitation was not documented in the present study. Thus, provided that the carious lesion was not pre-identified with certainty as being cavitated, at least 74.7% of FDSs would ‘drill and fill’ proximal enamel lesions (Fig 1), which could have benefited from non-invasive remineralisation strategies.^1^

Regarding the occlusal restorative threshold, 83% of the respondents would operatively intervene for lesions affecting the enamel and the outer third of dentin (Fig 2), for which therapeutic sealants would actually have been indicated.^32^ Moreover, for the earlier stage of caries progression requiring restoration, 15% of the respondents would perform a Black cavity preparation (opening the whole fissure system), which today would be considered iatrogenesis.^13^

The present results illustrate the presence of a gap between the evidence on the one hand and the knowledge and practices of FDSs on the other. Nevertheless, it seems that these FDSs tended to postpone invasive strategies to later stages of carious progression of occlusal lesions than FGPs did in 2012.^9^ Indeed, a substantially higher rate of surgical intervention by FGPs was observed for grade 2 occlusal carious lesions compared with FDSs (37.2% vs 9.6%), which clearly shows that FDSs were more likely to intervene at a later stage (72.3% at grade 3) than FGPs.^9^

Responses related to the two clinical cases (teeth 1 and 2) indicated that a statistically significant percentage of FDSs would operatively intervene when they diagnosed enamel lesions (34% and 51.6% for teeth 1 and 2, respectively) or even when they were uncertain of the presence/absence of a carious lesion (32.1% and 40% for teeth 1 and 2, respectively). This could reflect the fear of FDSs in the face of uncertainty, as already described among dentists.^2^ However, in a non-life-threatening context involving caries (in absence of specific medical conditions), over-treatment is iatrogenesis, and a conservative approach such as sealants is recommended for ICDAS 1-4 lesions, particularly in patients whose risk factors are under control, as suggested in the present survey.^30,32^ FDSs’ responses were based on the radiographs and photographs provided in the questionnaire. It can be argued that some clinical elements such as probing with the WHO probe or a precise evaluation using ICDAS might not be clear (especially the distinction between ICDAS 1 and 2, which are only presented on dry occlusal surfaces).

It seems that the reading of scientific articles positively influenced FDSs’ treatment decisions. Indeed, FDSs who read scientific articles were more likely to postpone their proximal restorative threshold for later stages of caries progression and more often opted for a minimally invasive cavity design such as tunnel preparation. French Dental Universities must encourage FDSs to improve their education through scientific literature, as only a minority (34.8%) reported reading scientific articles about MI in cariology. These findings are consistent with those of Hélie et al,^15^ who found that FGPs who read scientific articles on this topic were more likely to be convinced of the effectiveness of preventive sealants. Likewise, differences in responses were noted according to gender, which corresponds to studies conducted among FGPs.^12,15,33^

Some limitations of the present survey should not be overlooked. First, the present study was conducted among FDSs in their fifth year of the dental curriculum, which is the penultimate year before graduation. At the French Dental Universities, the last year is devoted to internships (similar to vocational training) in private practice and only management and professionally oriented courses are given; the certificate of practice is delivered at the end of the 5th year. Thus, a better integration of MI concepts could have been expected among the 6th-year FDSs and the start of the final year of training would surely have been the most appropriate time to administer the questionnaire. Secondly, it can be hypothesised that treatment attitudes in ‘real’ practice settings may differ from those reported in the present survey. Indeed, as this study was purely declarative, desirability bias cannot be excluded. Further, it may be difficult to extrapolate our findings to other countries due to the variety of dental practices and curricula. The multiplicity of analytical analyses could have led to false-positive results. Finally, reading scientific articles was not based on any specific criteria.

Improving FDSs’ knowledge and behaviours with respect to MI requires reinforcement of their training on this topic, to better adhere to recent curricula on cariology^3,5,29^ and consensus for carious lesion management.^31^ Besides, some FDSs who do not adhere to MI may in fact be insufficiently aware of MI, have mistaken beliefs or they consciously do not put into practice their knowledge for fear of missing a potential carious lesion that would necessitate surgical intervention. In the latter cases, the potential barriers or reasons for not using MI ought to be understood. Qualitative studies on these issues could provide additional explanations useful for optimising MI dissemination among these FDSs. In addition, it would be relevant to precisely reference the content of the cariology courses in the 16 French dental schools to highlight the potential gaps between the evidence of MI and the different dental curricula, as has been done in Australia, New Zealand, and the Spanish-speaking countries of Latin America.^21,27^ It should be noted that a cross-sectional comparison between faculties was not intended in this study, so as not to point out any discrepancies between individual teaching approaches/curricula, but rather to point out the need for standardisation. The aim of the CNEOC (French national association of teachers in conservative dentistry) is to finally promote the definition of a single curriculum agreed upon at the national level.

## Conclusion

There is a need to improve the awareness of FDSs about MI, to induce them to postpone invasive strategies to later stages of caries progression (lesion involving the middle third of dentin) and to prefer non-invasive or preventive strategies (topical fluoride application, therapeutic sealants and monitoring). Indeed, a more conservative approach in the management of carious lesion will avoid starting a deleterious cycle of dental restorations, thus improving the cost effectiveness of our treatment. Likewise, promotion of wider use of MI in dental practice by the National Guidelines is desirable and better reimbursement of preventive and restorative therapeutic treatment in dentistry is also needed.
